# Making food-related health taxes palatable in sub-Saharan Africa: lessons from Ghana

**DOI:** 10.1136/bmjgh-2023-012154

**Published:** 2023-10-09

**Authors:** Amos Laar, James M Amoah, Labram M Massawudu, Kingsley K A Pereko, Annabel Yeboah-Nkrumah, Gideon S Amevinya, Silver Nanema, Emmanuel Ankrah Odame, Percy A Agyekum, Mary Mpereh, Sebastian Sandaare, The Advocating for Health Coalition

**Affiliations:** 1 Department of Population, Family & Reproductive Health, University of Ghana School of Public Health, Accra, Greater Accra, Ghana; 2 Ghana Public Health Association, Accra, Ghana; 3 Ghana NCD Alliance, Accra, Ghana; 4 Ghana Academy of Nutrition and Dietetics, Cape Coast, Ghana; 5 Ghana Ministry of Health, Accra, Greater Accra, Ghana; 6 Korle-Bu Teaching Hospital, Accra, Ghana; 7 Food and Drug Authority Ghana, Accra, Greater Accra, Ghana; 8 National Development Planning Commission, Accra, Greater Accra, Ghana; 9 Parliament of Ghana, Accra, Accra, Ghana; 10 Coalition of Actors for Public Health Advocacy, Accra, Ghana

**Keywords:** public health, nutrition, health economics, health policies and all other topics

## Abstract

Amidst high burden of infectious diseases, undernutrition and micronutrient deficiencies, non-communicable diseases (NCDs) are predicted to become the leading cause of death in Ghana by 2030. NCDs are driven, to a large extent, by unhealthy food environments. Concerned, the Ghana Ministry of Health (MOH) has since 2012 sought to garner the support of all to address this challenge. We aimed to support the MOH to address the challenge through public health policy measures, but would soon be reminded that longstanding challenges to policy development such as data poverty, and policy inertia needed to be addressed. To do this, the we generated the needed evidence, curated the evidence, and availed the evidence to Ghanaian policymakers, researchers and civil society actors. Thus, we addressed the problem of data poverty using context-relevant research, and policy inertia through advocacy and scholar activism. In this paper, we share how a public interest coalition used context-relevant research, evidence-informed advocacy and scholar activism to valorise and increase demand for healthy food policy (including food-related health taxes) in Ghana.

**Summary box**

- We present the work of a public interest coalition as a best practice example of how coalition building, evidence-informed advocacy and scholar activism can valorise and increase demand for healthy food policies in Africa.
- To address longstanding challenges to policy development such as data poverty, and policy gradualism, investment in context-relevant evidence, appropriate curation of the evidence and availing the evidence to different actors (policymakers, researchers, civil society actors and lay public) are not desiderata but requirements.
- This example is not a magic bullet, but we believe that well thought-out coalitions that are armed with context-relevant data, and capacitated to advocate for the health of their countries may increase uptake of the policies in their jurisdictions.
- Public health advocates have to be willing and ready to counter and debunk oppositional arguments from actors with vested interests.
- Advocates and policymakers need to appreciate the peculiar heterogeneity of the African food environments and its variegated political economies.

## Introduction

### General context

Located on the west coast of Africa, the Republic of Ghana has a population of about 32 million people; majority of whom live in urban areas where the food environments are rapidly changing. In 2015, Ghana achieved the Millennium Development Goal 1 Target 1C – of *halving, between 1990 and 2015, the proportion of people who suffer from hunger*. Ghana is currently on course to achieve the stunting-related sub-target of Sustainable Development Goal 2, which is to achieve a 5% reduction in the number of stunted children by 2025. Life expectancy at birth is 61 years for men and 64 years for women. Mortality among children under-5 years and women of reproductive age has declined steadily over the last two decades but still remains unacceptably high. A high burden of infectious disease and malnutrition in all its forms (undernutrition, micronutrient deficiencies and an emerging epidemic of diet-related non-communicable diseases (NCDs) are the key drivers of mortality.

### Public health nutrition context

In the past two decades, Ghana has witnessed significant improvements in childhood undernutrition. Among children under-5 years, stunting rate (indicative of chronic undernutrition) has declined from 28% in 2008 to a current rate of 19% in 2014; acute undernutrition (wasting) has been halved to the current 5%. Among adult women of reproductive age, anaemia rates have declined from about 60% in 2008 to 42% in 2014.^1^ Notwithstanding such reductions, the current rates of anaemia in both women (42%) and children under-5 (66%) remain above acceptable public health thresholds. Data from the Ghana Demographic and Health Survey show that overweight and obesity rates among adult women have skyrocketed from 10% in 1993 to 40% in 2014.^1^ The rates are higher in urban areas where the food environments are rapidly changing. There is a relatively low but increasing rate of obesity among children <5 years—3%^1^ and 17% among children aged 9–15 years old and living in urban Ghana.^2^ Other local studies report a very high prevalence of overweight/obesity among Ghanaian children^2–4^ and adults.^5^ Ranging from 16%^2 3^ to 46%^4^—for children, and 25% to 47% for adults.^5^

It has been estimated that over one-third of all adult deaths are due to NCDs^6^ while the risk of premature death from select NCDs is 15%.^7^ Such increases in rates of other diet-related NCDs such as hypertension, diabetes and cardiovascular disease^7^ amidst undernutrition and chronic food insecurity means that Ghana is experiencing multiple burdens of malnutrition. While the causes of overweight, obesity and other diet-related NCDs are complex and with multiple interacting determinants, dietary factors such as excessive consumption of calorie-dense nutrient-poor foods are paramount. Urgent actions are needed, as the economic, and public health impacts of inaction are incalculable.^6 8–10^

### Policy context

Since Ghana became independent in 1957, there has been demonstrated political commitments to address malnutrition, particularly food insecurity and undernutrition. Local efforts to address food insecurity and undernutrition include the development of various legislations, regulations, policies, strategies and programmes.^11 12^ Significant efforts to address nutrition-related NCDs did not begin until the past decade. In 2012, the Ministry of Health (MOH) published the first ever national NCDs policy and accompanying strategy.^7^ The 2022 version of the policy refers to interventions (including regulating advertisement of unhealthy foods and non-alcoholic beverages, particularly to children), limiting the level of trans fats and salt in industrially processed food as well as food-related health taxes.^13^ Other ministries, departments and agencies (MDAs) have shown their support to addressing the problem of NCDs. In 2019, an interministerial dialogue produced a government ‘consensus statement’ that acknowledged the value of improving the Ghanaian food environments to deliver healthy diets and better nutrition. Participating in the dialogue, H.E., First Lady of the Republic of Ghana, called for a paradigm shift that repositions the Ghanaian food systems from ‘feeding’’ the population to ‘nourishing’’ them. In 2021, H.E., the President of the Republic of Ghana, expressed Ghana’s commitment to transforming its food systems by 2030, so as to assure sustainable healthy diets. Among several targets to be achieved, are *development and implementation of food-based dietary guidelines by 2022; updating, and consolidating local food composition databases; and development of a nutrient profiling system to facilitate implementation of several food-based policies*.

Currently available, although with sparse implementation (particularly in Africa), are several evidence-based interventions that can reduce the burden of NCDs among populations. These include the WHO *Best Buys* 14 (eg, increasing excise taxes and prices on tobacco products, and on alcoholic beverages, front-of-pack labelling of food products), so-called due to their cost-effectiveness and feasibility for combating NCDs in low and middle income countries. Reducing sugar consumption through effective taxation on sugar-sweetened beverages (SSB) is considered an effective intervention.^14 15^ In Ghana, stakeholders across multiple agencies have endorsed the WHO *Best Buys* for controlling exposure to harmful products such as alcohol, tobacco and unhealthy diets.^12^ In 2004, Ghana ratified the WHO Framework Convention on Tobacco Control.^16^ Subsequently advertising bans for tobacco were implemented, as well as an increase in tobacco taxes, and designation of limited smoke-free locations (including government buildings and vehicles and other public spaces like hotels). In 2017, a National Alcohol Policy^17^ was launched to regulate production, distribution, sale, advertisement and consumption of alcohol. The policy identified priority strategies for the reduction of alcohol-related harms. These strategies included taxation, regulating availability and marketing of alcohol.

Tax-related NCD prevention interventions have existed for tobacco (since 2004) and alcohol (since 2017). Those linked to unhealthy diets such as SSB tax was recently introduced (in April 2023). Currently, the following are some of the regulations/laws governing administration of excise duty in Ghana. Excise Duty Act, 2014 (Act 878); Excise tax Stamp Act, 2013 (Act 873); Excise Duty Regulations, 2016 (L.I. 2242); Excise Tax Stamp Regulations 2016, (L.I. 2241); Revenue Administration Act, 2016 (Act 915); and Excise Duty Amendment Act (Act 1093).

Due to their potential to improve population health and raise revenue, there is significant and growing interest in SSB taxes among policymakers worldwide. Focusing on just SSB taxes, the WHO reported that some 85 countries and jurisdictions (including subnational levels) have levied taxes on SSBs.^18^ Recently the World Bank Group report that SSB taxes are in effect across all World Bank regions, including national level taxes in more than 100 countries and territories.^19^ Although there is considerable global evidence on the positive impact of fiscal interventions (including food-related health taxes) on public health,^15 20^ implementation of such interventions in Africa is currently sparse.^12 15 19 21^ The African countries that have enacted SSB taxes include South Africa, Mauritius, Seychelles, Morocco, Botswana, Nigeria, and recently Ghana. The policy has been implemented and evaluated in several countries in Europe, Latin American and the Caribbean. In 2014, Mexico introduced a tax of 10% or 1-peso-per-litre tax on beverages containing added sugar. An analysis of Mexican sugary beverage sales showed about 6% reduction in purchased volume relative to pre-tax trends over the first year of the tax, and a 9.7% decrease in 2015.^22^ The largest decrease in purchases was among the most socioeconomically disadvantaged. Implemented since October 2014, the Chilean SSB tax (18% ad valorem tax on sugary drinks containing >6.25 g sugar/100 mL) was able to reduce the monthly purchased volume of the higher taxed, sugary soft drinks by 21.6%.^23^

Our Coalition aimed to valorise and increase demand for these policies through advocacy and scholar activism. The Coalition’s motivations, ideology and goals are briefly outlined. The Coalition was formed to contribute to efforts that address the high and rising burden of undernutrition, NCDs in Ghana. Aware that data poverty and policy inertia present critical challenges, the coalescing of the efforts of academia, civil society organisations and public health associations, was required to address these problems. The Coalition aimed to address data poverty through research and evidence synthesis, and policy inertia through advocacy and sensitisation. Members of the Coalition have similar policy belief systems and philosophy, which is:

If the government of Ghana implements comprehensive policy measures - a mix of low agency and high agency food environment policy measures to inform and empower consumers; to guide and influence consumers; to incentivize the consumption of healthier foods, and to discourage/ disincentivize consumption of unhealthy foods, then food actors (eg, producers and consumers) will make immediate or strategic decisions to reduce availability, attractiveness and consumption of such less healthy foods, or increase availability, attractiveness, and consumption of healthier foods.

Given its manifold applications, a nutrient profiling model (NPM) was chosen as a tool to facilitate the implementation of these policies. The numerous applications of the NPM in the food systems (eg, agricultural policies; international trade policy; food manufacturers and processors; catering in healthcare facilities for patients, staff and visitors; in food marketing and promotion, packaging and labelling; in school food environment; fiscal policies (eg, taxes, and subsidies) have been detailed elsewhere (See McColl *et al*). Of note, to effectively assess the impact of the policies, existing data and monitoring gaps need to be addressed.

## The strategies and approaches we used

We deployed multiple strategies including coalition building, action research, advocacy and scholar activism. We list herein, and briefly outline, 10 of these strategies.

1. Coalition building
2. Contextual analysis and evidence synthesis.
3. Strategic planning.
4. Development of advocacy strategy and a communication plan.
5. Public mobilisation and sensitisation.
6. Issuance of position statements to key actors.
7. Distribution of press statements to relevant actors.
8. Press conferences.
9. Stakeholder engagements and dissemination.
10. Social media, blogging and op-eds.

### Overview of the strategies and approaches we deployed

**Figure 1:**
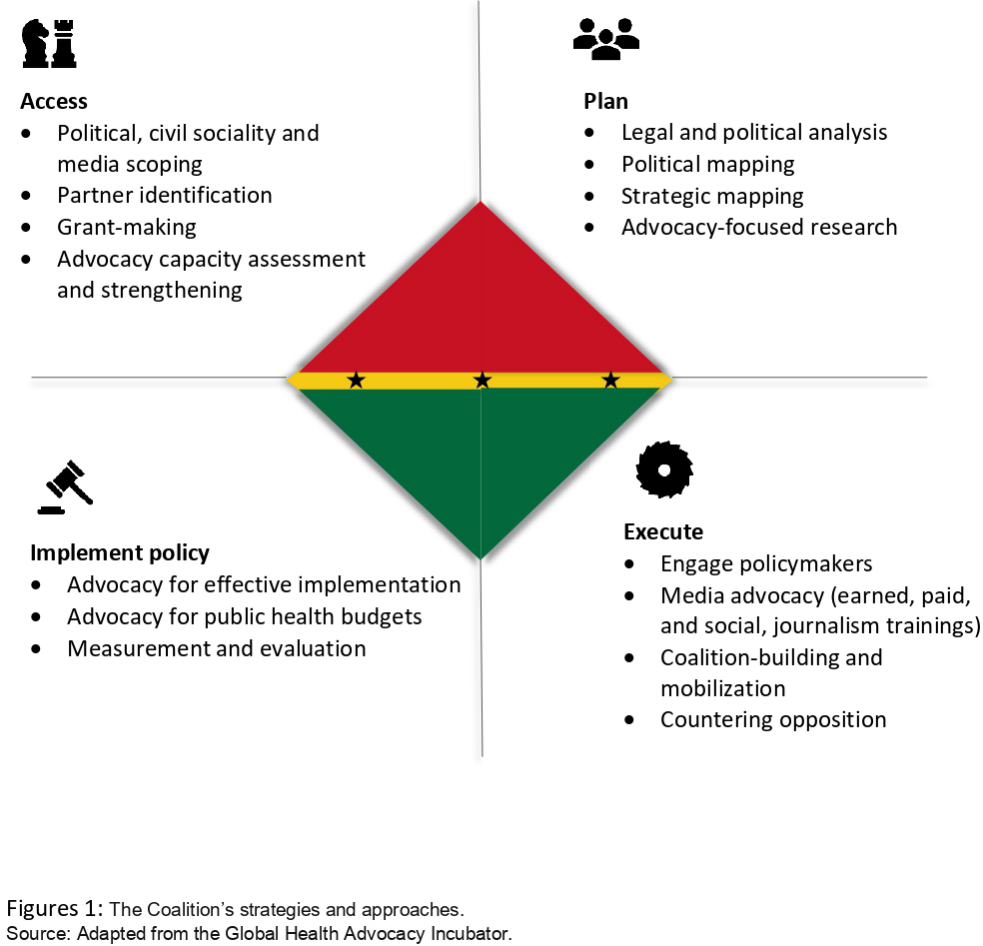
The Coalition’s strategies and approaches. Source: The Global Health Advocacy Incubator.

**Figure 2:**
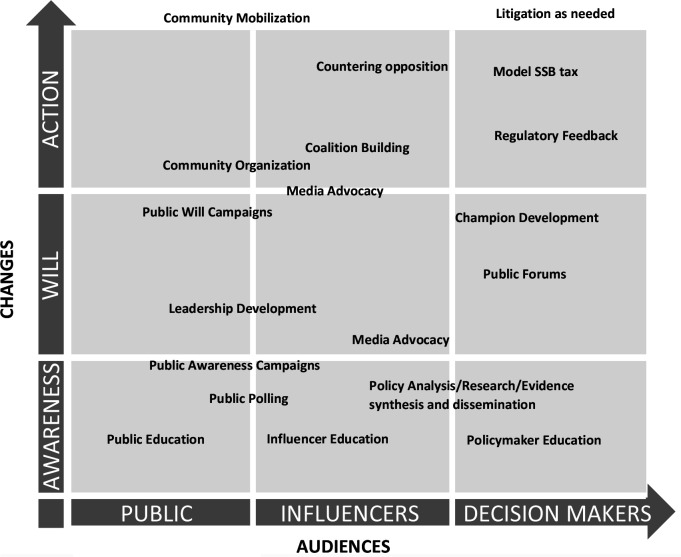
Select advocacy strategies, audiences and the changes desired. Source: Coffman and Beer.^25^

With technical support from the Global Health Advocacy Incubator (GHAI), our strategies borrowed significantly from their ‘path to policy change’ framework.^24^ The strategy began with data generation/landscaping (including legal feasibility analysis and political mapping), evidence/best practice synthesis. This was followed by strategic planning; advocacy execution and ended with policy evaluation (see figure 1). The advocacy work included capacity building, sensitisation and awareness creation; strategic communications, media advocacy and policy advocacy. The framework that undergirded the advocacy intervention was developed by Coffman and Beer.^25^ Our chosen strategies were informed by our target audiences (public, influencers and decision-makers)—see figure 2.

#### Coalition building

We built a coalition whose primary goal is to contribute to improving Ghana’s health. A public interest coalition that brings together both state and non-state actors as well as existing networks. Referred to as the Advocating for Health (A4H) Coalition, it comprises—The A4H Project, the ‘Healthier Diets 4 Healthy Lives (HD4HL) Project, Academics from five public universities of Ghana (led by the School of Public Health, University of Ghana), Civil Society Organizations (including the Ghana NCD Alliance), Public Health Associations (eg, the Ghana Public Health Association), Nutrition/Dietetic Professional Group (Ghana Academy of Nutrition and Dietetics) and others, for example, the Coalition of Actors for Public Health Advocacy (CAPHA). With funding from the IDRC, Canada, The Rockefeller Foundation, Bloomberg Philanthropies and technical (advocacy) support from the GHAI, the A4H Coalition, among others seeks to create—in Ghana—a favourable environment and stakeholder buy-in for food-related health taxes (which includes taxes on SSBs). Of note, members of the Coalition share similar policy belief systems and engage in a coordinated approach to promote their position and achieve the ultimate goal of improving Ghana’s food environments and addressing the NCD challenge.

It is common knowledge that the design and implementation of SSB tax policies are beset with many challenges. Local economies, but also, the power asymmetries between ‘public’ and ‘private’ interests confound promulgation and implementation of these policies. Morocco had a very challenging journey—having to repeal its SSB tax passed in 2018 prior to implementation in 2019—in response to pressures from the agri-food industry.^26^ The revised version watered down. Similar watering down experiences have been reported in South Africa and Nigeria. We devised guardrails to confront industry opposition and interference as well as conflicts of interests of the coalition actors. Led by the ‘Healthier Diets for Healthy Lives (HD4HL) Project’, the engagement of stakeholders was preceded by an onboarding guidance on the conflict of interest; all stakeholders signed a conflict of interest declaration form. Quite recently, the Coalition members received a full-day training on corporate political activity and industry interference in public health research and policy. The training made significant references to the recently published series on Commercial Determinants of Health.^27 28^ The Coalition also pre-emptively prepared informed-informed rebuttals of common industry oppositional arguments.

##### About the HD4HL project, the A4H project and the A4H Coalition

Titled, ‘developing evidence and action towards a double-duty food-based policy bundle to assure healthier diets in Ghana’, the ‘HD4HL Project aims to develop, validate and build consensus for a fit-for-local-purpose NPM that allows for the implementation of food-based policies. The HD4HL Project, is itself a coalition, comprises government agencies (MoH, Food and Drugs Authority, National Development Planning Commission), academia (University of Ghana) and civil society organisations (CAPHA). See details in figure 1 and other details at the Project website—https://www.hd4hl.org/.

The A4H Project, a constituent of the A4H Coalition, aims to create a favourable environment and stakeholder buy-in for food-related fiscal policies (particularly SSB tax) in Ghana. This is achieved through evidence-informed advocacy and scholar activism. See details at the Project website—https://www.advocating4health.org/

#### Evidence synthesis

The Coalition’s use of action research and evidence synthesis was not an afterthought. Desirous of contributing to addressing the emerging NCDs challenge with the needed urgency, the Coalition prioritised public health policy interventions but would soon be reminded that longstanding challenges to policy development including data poverty, and policy inertia, needed to be addressed. Mindful of the notion that “doing the wrong thing at the right time is wrong, and doing the right thing at the wrong time is equally wrong”, the Coalition took a step back, and had the academic partners channel their energies toward generating evidence, curating the evidence and availing the evidence to relevant actors and stakeholders (including researchers, policymakers and civil society).

To generate the evidence, we engaged people in their communities and assessed dietary practices, food availability and marketing in the communities and neighbourhoods. We also engaged relevant national-level food environment stakeholders to identify priorities for national action. Through dietary intake and time use study, we assessed how unhealthy food and beverages are embedded in everyday life.^29^ We used novel qualitative methods to identify factors shaping dietary behaviours^30^ and geo-mapping techniques to show how unhealthy food is advertised and sold through.^31^ Our television monitoring, outdoor food advertising assessment and supermarket assessment revealed heavy marketing and widespread availability of unhealthy foods.^32–34^ The evidence informed the development of policy recommendations to improve the healthiness of the Ghanaian food environment. The recommendations included development of policies that account for people’s lived environments and encompass regulatory, legislative and fiscal measures.

But are Ghanaians ready for these interventions? Our community readiness assessment helped gauge how ready Ghanaian communities are to accept and implement those policies.^35^ Also, a nationwide poll involving a sample of 7794 residents of Ghana provided policymakers and other stakeholders with the needed local evidence to gauge public support for such policies. For example, data from the poll revealed that a significant number of Ghanaian residents (84.3%) were concerned about obesity and NCDs (in adults and children). Two out of three respondents (67.7%) would support any effort by the government to impose tax on health-harming commodities and products (such as SSBs). When respondents were told the money from the taxes could be used to support public health interventions, support for the tax increased to nearly 80% (77.4%).^36^

We knew that the empirical evidence generation alone was not enough. As part of our contextual analysis, we conducted a legal feasibility analysis (a legal landscaping to identify a realistic legal pathway to enact SSB tax, and other healthy food policies in Ghana), we conducted political economy analysis (examining the roles of politics, economics and institutions in policy decisions), corporate political activity analysis (analysing the commercial determinants of health potential impact of private actors on the policy development) as well as policy coherence analysis (where existing national nutrition and related policies were examined to gauge policy relevant and to avoid policy conflicts). The legal feasibility analysis, for instance, revealed that ‘there was currently no SSB tax law in Ghana, although an excise tax exists for multiple commodities, including SSBs exist. The existing laws in Ghana make it legally feasible to adopt an SSB tax in Ghana—as a health tax. The political will and desire to introduce SSB tax law was evidenced by the government of Ghana’s (led by the MoH) declaration to develop a bundle of food-based policies (including front-of-pack labelling, marketing regulations and food-related fiscal policies). The Coalition also commissioned several rapid systematic reviews. Box 1 summarises the various contextual analysis and evidence synthesis that was carried out by the Coalition.

Box 1 **Contextual analysis and evidence synthesis implemented by the coalition**

1. Legal feasibility analysis—analysis of the legal framework under which the policies (eg, SSB Tax, FOPL, Marketing restrictions) would be introduced.
2. Political economy analysis of the proposed policies (examination of the politico-economic dynamics).
3. Corporate political activity analysis of the proposed policies.
4. Policy coherence analysis—analysis of existing national nutrition and related policies, to examine the feasibility of implementation and the appropriateness of the timing for development.
5. The macroeconomic impacts of diet-related fiscal policy: a rapid review.
6. The impact of a sugar-sweetened beverage tax on selected health outcomes: a rapid review.
7. Implementation of a sugar-sweetened beverage tax in low and middle-income countries: documenting the processes, best practices, challenges, etc) and recommendations for Ghanaian policymakers.
8. Implementation of front-of-pack labelling in low and middle-income countries: documenting the processes, best practices, challenges, etc) and recommendations for Ghanaian policymakers.
9. Implementation of marketing regulations in low and middle-income countries: documenting the processes, best practices, challenges, etc) and recommendations for Ghanaian policymakers.
10. Implementation of public food procumbent policies in low and middle-income countries: documenting the processes, best practices, challenges, etc) and recommendations for Ghanaian policymakers.
11. Implementation of nutrient profiling systems in low and middle-income countries: documenting the processes, best practices, challenges, etc) and recommendations for Ghanaian policymakers.
12. The potential impact of a sugar-sweetened beverage tax on revenue: an economic modelling analysis.
13. The state of Ghana’s food composition databases.
14. Epidemiological analysis (desk review of local evidence): diet-related NCDs and the population’s dietary patterns (prevailing intake levels of nutrients to encourage or limit, prevalence of inadequacies in intakes of key positive nutrients and excess diet-related NCDs, micronutrient malnutrition).

FOP, front of pack labelling; NCD, non-communicable disease; SSB, sugar-sweetened beverage.

#### Strategic planning

Toward realising the goals of the Coalition, for example, the goals, expected outcome, strategic interventions, key tasks and indicators to track were generated and included in a strategic plan. These facilitated strategic decision-making and actions of the Coalition.

#### Development of an advocacy strategy and a communication plan

We developed an advocacy strategy, and a communication plan to facilitate our advocacy and communication. In particular, the communication plan provided a framework for communicating the project’s actions by the partnering institutions to all identified audiences; to build awareness and understanding of Coalition goals and impacts; help promptly address anticipated and emerging issues and concerns that have the potential to derail the goals of the Coalition, communicate promptly any problems affecting the Coalition.

#### Mobilisation of allies, sensitisation of the public and advocacy interventions

A mapping of relevant actors (particularly allies) preceding the coalition’s sensitisation activities. Through floats, public fora, route marches and media advocacy, we mobilised and sensitised residents of Ghana about the harms of SSBs, and the need for health-harming foods to be taxed. These activities, together with our blog posts and op-eds resulted in about 100 media coverage/reportage (see details in table 1). We also identified influential personalities as advocacy Champions and Influencers to help push the Coalition’s messages to the last mile.

**Table 1:** Advocating for health project media report (July 2022–February 2023)

2a News (digital and press)		
**Date**	**Title of news articles and project-related activity**	**URL and media house**
24 Jul 2022	‘Sugar-sweetened beverages increase risk of Type 2 diabetes’ **Float in Tamale**	**Graphic Online:** https://www.graphic.com.gh/lifestyle/sugar-sweetened-beverages -increase-risk-of-type-2-diabetes.html
22 Jul 2022	CSOs sensitize public on sugar sweetened beverages **Float in Tamale**	**Ghana News Agency**: https://gna.org.gh/2022/07/csos-sensitise-public-on-sugar-sweetened-beverages /
28 Oct 2022	Public Health Ghana advocates for tax increment on sugary products **Float in Kumasi**	**Graphic Online**: https://www.graphic.com.gh/lifestyle/public-health-ghana-advocates-for-tax-increment-on-sugary-products.html
26 Oct 2022	Increase taxes on sugary beverages—GPHA urges Government **Float in Kumasi**	**Ghana News Agency:** https://gna.org.gh/2022/10/increase-taxes-on-sugary-beverages-gpha-urges-government/
28 Oct 2022	Increase taxes on sugary beverages—GPHA urges Government **Float in Kumasi**	**Business Ghana:** https://www.businessghana.com/site/news/general/273361/Increase-taxes-on-sugary-beverages-GPHA-urges-government
27 Oct 2022	GPHA wants gov’t increase taxes on sugary beverages **Float in Kumasi**	**The Chronicle:** https://thechronicle.com.gh/gpha-wants-govt-increase-taxes-on-sugary-beverages/
28 Oct 22	GPHA calls for a 20% tax increment on sugary beverages **Float in Kumasi**	**The Independent Ghana:** https://theindependentghana.com/gpha-calls-for-a-20-tax-increment-on-sugary-beverages/
28 Oct 2022	Sugar-sweetened beverages kill; stop patronizing them—Nutritionist **Float in Ho**	**My Joy Online:** https://www.myjoyonline.com/sugar-sweetened-beverages -kill-stop-patronising-them-nutritionist/
27 Oct 2022	Ghanaians urged to reduce intake of sugar sweetened beverages **Float in Ho**	**Ghana News Agency:** https://gna.org.gh/2022/10/ghanaians-urged-to-reduce-intake-of-sugar-sweetened-beverages /
28 Oct 2022	Ghanaians urged to reduce intake of sugar sweetened beverages **Float in Ho**	**GBC Online**: https://www.gbcghanaonline.com/uncategorized/sugar-zotor/2022/
27 Oct 2022	Reduce the intake of sugar sweetened beverages **Float in Ho**	**News Ghana:** https://newsghana.com.gh/reduce-the-intake-of-sugar-sweetened-beverages /
29 Oct 2022	Impose heavy taxes on Sugar-Sweetened Beverages to make them unattractive- GhNCDA **Journalist Sensitization**	**My Daily News:** https://mydailynewsonline.com/impose-heavy-taxes-on-sugar-sweetened-beverages -to-make-them-unattractive-ghncda/ **News Ghana**: https://newsghana.com.gh/impose-heavy-taxes-on-sugar-sweetened-beverages -to-make-them-unattractive-ghncda/
31 Oct 2022	Health Taxes: GhNCDA wants Sugar Sweetened Beverages taxed **Journalist Sensitization**	**Norvan Reports:** https://norvanreports.com/health-taxes-ghncda-wants-sugar-sweetened-beverages -taxed/
31 Oct 2022	Ghana needs sugar-sweetened beverages tax for health financing—CSOs **Journalist Sensitization**	**Ghana News Agency**: https://gna.org.gh/2022/10/ghana-needs-sugar-sweetened-beverages -tax-for-health-financing-csos/
01 Nov 2022	‘Increase Taxes on Sugar Beverages’ **Journalist Sensitization**	**Daily Guide Network:** https://dailyguidenetwork.com/increase-taxes-on-sugar-beverages/
31 Oct 2022	Non-communicable diseases to be leading cause of death in Ghana by 2030 **Journalist Sensitization**	**Metro TV Online:** https://metrotvonline.com/non-communicable-diseases-to-be-leading-cause-of-death-in-ghana-by-2030-prof-laar/ **My Original Online:** https://myoriginalonline.com/blog/non-communicable-diseases-to-be-leading-cause-of-death-in-ghana-by-2030-prof-laar/
01 Nov 2022	Increase taxes on Sugar Beverages **Journalist Sensitization**	**Daily Guide (print; not included**)
10 Nov 2022	Government urged to place taxes on SSBs to curb consumption **Public Forum in Kumasi**	**My Joy Online:** https://www.myjoyonline.com/government-urged-to-place-taxes-on-ssbs-to-curb-consumption/
16 Nov 2022	Soaring health costs, deaths linked to diet-related NCDs alarming—A4H Coalition **Float in Accra**	**My Original Online:** https://myoriginalonline.com/blog/soaring-health-costs-deaths-linked-to-diet-related-ncds-alarming-a4h-coalition/
16 Nov 2022	Soaring health costs, deaths linked to diet-related NCDS alarming—A4H coalition **Float in Accra**	**Metro TV Online:** https://metrotvonline.com/soaring-health-costs-deaths-linked-to-diet-related-ncds-alarming-a4h-coalition/
16 Nov 2022	Soaring health costs, deaths linked to diet-related NCDs alarming—A4H Coalition **Float in Accra**	**Ghana web:** https://www.ghanaweb.com/vip/ernestsenanudovlo/Soaring-health-costs-deaths-linked-to-diet-related-NCDs-alarming-A4H-Coalition-23519
08 Feb 2023	A4H & HD4HL coalition applaud government’s fiscal policy approach to addressing unhealthy diets **Joint Position statement (HD4HL & A4H PROJECT Coalition**)	**Catholic Trends:** https://catholic-trends.com/2023/02/08/a4hhd4hl-coalition-applaud-governments-fiscal-policy-approach-to-addressing-unhealthy-diets/ **Ghana Web:** https://www.ghanaweb.com/vip/ernestsenanudovlo/A4H-HD4HL-coalition-applaud-government-s-fiscal-policy-approach-to-addressing-unhealthy-diets-51047 **My Original Online:** https://myoriginalonline.com/blog/a4hhd4hl-coalition-applaud-governments-fiscal-policy-approach-to-addressing-unhealthy-diets/ **Metro TV Online:** https://metrotvonline.com/a4hhd4hl-coalition-applaud-governments-fiscal-policy-approach-to-addressing-unhealthy-diets/
10 Feb 2023	Taxation of Sugar-Sweetened Beverages is a Win-Win-Win Strategy for Public Health, for Government Revenue, and for Health Equity **Press statement release by A4H Coalition**	**Project webpage** https://www.advocating4health.org/press-release-taxation-of-sugar-sweetened-beverages / **Metro TV Online:** https://metrotvonline.com/true-economic-health-costs-of-ssbs-staggering-a4h-coalition/ **My Original Online:** https://myoriginalonline.com/blog/a4h-ghana-commends-government-for-its-intended-taxation-of-sugar-sweetened-beverages / **Ghana Web:** https://www.ghanaweb.com/vip/ernestsenanudovlo/Taxing-sugar-sweetened-beverages -a-win-win-win-strategy-for-Ghana-A4H-Coalition-54767 **Mx24 Online:** https://mx24online.com/advocating-for-health-coalition-throws-support-behind-govts-proposal-to-tax-sugar-sweetened-beverages / **Ghanaian times:** https://www.ghanaiantimes.com.gh/taxation-of-sugar-sweetened-beverages -is-a-win-win-win-strategy-for-public-health-for-government-revenue-and-for-health-equity/
22 Feb 2023	Advocating for Health Coalition lauds govt’s proposal to tax sugar-sweetened beverages **Press Conference**	**Starr FM**: https://starrfm.com.gh/2023/02/advocating-for-health-coalition-lauds-govts-proposal-to-tax-sugar-sweetened-beverages /
17 Feb 2023	Coalition advocates imposition of taxes on tobacco, alcohol, sugary foods **Press Conference**	**Ghana news agency**: https://gna.org.gh/2023/02/coalition-advocates-imposition-of-taxes-on-tobacco-alcohol-sugary-foods/
17 Feb 2023	NCDs predicted to become the leading cause of death in Africa **Press Conference**	**Capital news**: https://capitalnewsonline.com/ncds-predicted-to-become-the-leading-cause-of-death-in-africa/
17 Feb 2023	Implement comprehensive policy measures to make unhealthy foods unattractive **Press Conference**	**News Ghana**: https://newsghana.com.gh/implement-comprehensive-policy-measures-to-make-unhealthy-foods-unattractive/
17 Feb 2023	Tax health-harming commodities to save lives **Press Conference**	**My daily news online**: https://mydailynewsonline.com/tax-health-harming-commodities-to-save-lives/
18^th^ Feb 23	Nation records surge in diet related non-communicable diseases **Press Conference**	**Daily Graphic** (print; not included)
**2b Audio-visual news**		
**Date**	**Title of video and project-related activity**	**URL and Media house**
25 Jul 2022	GPHA leads Advocating for Health (A4H) to raise awareness on SSBs in Tamale—Sagani TV Mid-day news **Tamale Float**	**Sangani TV English** https://www.youtube.com/watch?v=lI9r5b4eS4w&t=6s
26 Jul 2022	Advocating for Health organizes float on SSBs—Sagani TV News in Dagbani **Tamale Float**	**Sangani TV Dagbaani** https://youtu.be/6SlAWIv44jg
16 Aug 2022	Sugar Sweetened Beverages are harmful to your health **Tamale Float**	**Citi News** https://www.youtube.com/watch?v=H9EVayT710s
26 Oct 2022	GPHA float on SSBs in Kumasi—Kessben Fm and TV news report **Kumasi Float**	K**essben TV** https://www.youtube.com/watch?v=mNVUtgZHXEA&t=60s
26 Oct 2022	GPHA Creates awareness on Sugar Sweetened Beverages in Kumasi—Onua TV News **Kumasi Float**	**Onua TV** https://www.youtube.com/watch?v=ydQkIvrj1Qs&t=8s
22 Nov 2022	A4H organizes float in Accra to create awareness on SSBs—UTV News coverage **Accra Float**	**UTV** https://youtu.be/psZy93kBHrg
22 Nov 2022	A4H organizes float in Accra to create awareness on SSBs—TV3 News coverage **Accra Float**	**TV3** https://youtu.be/1j6WcrY8U7A
31 Oct 2022	Sensitization Workshop for Journalist on Public Health Implications of Sugar Sweetened Beverages **Journalist Sensitization**	**Morris TV GH** https://www.youtube.com/watch?v=51lcfnBo6JY
8 Nov 20 922	GhNCDA urges Gov’t to place heavy taxes on foods to reduce intake and health implication rate **Public Forum**	**Mx24 TV** https://drive.google.com/drive/folders/1luxhc2Y1MeyhMMvC_gSkhWc8EfsFXmSw?usp=sharing
16 Feb 2023	Taxation of sugar sweetened beverages **Press Conference**	**Mx24 TV** (Start from 53 mins) https://www.facebook.com/mx24gh/videos/725720492295171/?vh=e&mibextid=v7YzmG
17 Feb 2023	Sugar sweetened beverages—causes of cardiovascular diseases **Press Conference**	**GTV (Start from 42 mins**) https://fb.watch/iMNUMoNDLL/
17 Feb 2023	Excessive sugar intake; Group calls for taxation of sugar-sweetened beverages to curb deaths **Press Conference**	**Adom TV** https://fb.watch/iLbsQmWLEV/
16 Feb 2023	Experts call for imposition of more taxes on ‘destructive commodities’ **Press Conference**	**Metro TV (Start from 45 mins**) https://fb.watch/iKdWXFxPe0/
17 Feb 2023	Excise Duty Amendment Bill; Advocating for Health Coalition urges Government to speed up its process **Press Conference**	TV Africa start from third mins) https://m.facebook.com/story.php?story_fbid=2246758068857525&id=100063970033275&mibextid=NnVzG8
17 Feb 2023	Tax on Sugar sweetened beverages is a win-win strategy **Press Conference**	GhOne TV: (Start from 43 mins 44 s) https://fb.watch/iP18OdCZYw/?mibextid=5zvaxg
	**Compilation of TV reportage of Press Conference**	https://drive.google.com/drive/folders/1oknZqfZ-lpn1TCCWhxUgvnJpfh4otmBV?usp=sharing
**2c News paper articles**		
**Date**	**Title of news articles and author**	**URL and Media house**
3 Oct 22	Sugar-sweetened beverages—the silent killers **Prof. Francis Zotor**	**Graphic Online:** https://www.graphic.com.gh/features/features/sugar-sweetened-beverages -the-silent-killers.html
31 Oct 22	Ghana’s young population ‘snacking’ their way into diabetes **Senanu Dovlo**	**My Original Online:** https://myoriginalonline.com/blog/ghanas-young-population-snacking-their-way-into-diabetes/ **Ghana Web:** https://www.ghanaweb.com/vip/ernestsenanudovlo/Ghana-s-young-population-snacking-their-way-into-diabetes-18929 **Metro TV Online:** https://metrotvonline.com/ghanas-young-population-snacking-their-way-into-diabetes/
16 Dec 22	Protect kids from sugary drinks; Stop, rethink and take action now! **Annabel Yeboah - Nkrumah**	**Metro TV Online**: https://metrotvonline.com/protect-kids-from-sugary-drinks-stop-rethink-and-take-action-now/ **My Original Online:** https://myoriginalonline.com/blog/protect-kids-from-sugary-drinks-stop-rethink-and-take-action-now/
16 Dec 22	We need to be heard! A call for government to curb sugar-sweetened beverages menace **Selina Tobil**	**Metro TV Online:** https://metrotvonline.com/we-need-to-be-heard-a-call-for-government-to-curb-sugar-sweetened-beverages -menace/ **My Original Online:** https://myoriginalonline.com/blog/we-need-to-be-heard-a-call-for-government-to-curb-sugar-sweetened-beverages -menace/
25 Dec 22	My take on the excise duty amendment bill, 2022 **Prof. Amos Laar**	**Metro TV Online:** https://metrotvonline.com/my-take-on-the-excise-duty-amendment-bill-2022/ **My Original Online:** https://myoriginalonline.com/blog/my-take-on-the-excise-duty-amendment-bill-2022/ **Ghana Web:** https://www.ghanaweb.com/vip/ernestsenanudovlo/My-take-on-the-Excise-Duty-Amendment-Bill-2022-35369
28 Dec 22	Health costs and deaths linked to diet-related Non-Communicable Diseases mount **Prof. Amos Laar**	**My Joy Online:** https://www.myjoyonline.com/amos-laar-health-costs-and-deaths-linked-to-diet-related-non-communicable-diseases-mount/
16 Jan 2023	Yes! Taxation of sugar-sweetened beverages is a ‘Win-Win-Win’ Public Health Intervention **Annabel Yeboah - Nkrumah**	**My Original Online:** https://myoriginalonline.com/blog/yes-taxation-of-sugar-sweetened-beverages -is-a-win-win-win-public-health-intervention/ **Metro TV Online:** https://metrotvonline.com/yes-taxation-of-sugar-sweetened-beverages -is-a-win-win-win-public-health-intervention/ **My Joy Online:** https://www.myjoyonline.com/yes-taxation-of-sugar-sweetened-beverages -is-a-win-win-win-public-health-intervention/ **Mx24 Online:** https://mx24online.com/article-yes-taxation-of-sugar-sweetened-beverages -is-a-win-win-win-public-health-intervention/
**2d Media coverage related to the HD4HL Project**		
**Date**	**Title of News Article**	**URL and Media house**
23 Mar 2022	Professor Amos Laar and Team Awarded CA$1 225 000 Grant For ‘Healthier Diets 4 Healthier Lives’ Project	**University of Ghana** https://www.ug.edu.gh/news/professor-amos-laar-and-team-awarded-ca1225000-grant-%E2%80%98healthier-diets-4-healthier-lives%E2%80%99
11 Mar 2022	Healthier Diets for Healthy Lives Project launched in Accra*	**Project webpage** https://www.hd4hl.org/healthier-diets-for-healthy-lives-project-launched-in-accra/
16 Mar 2022	All you need to know about Ghana’s policy bundle initiative on healthier diets*	**Project webpage** https://www.hd4hl.org/all-you-need-to-know-about-ghanas-policy-bundle-initiative-on-healthier-diets/
3 Apr 2022	Prof. Amos Laar lauded for Healthier Diets for Healthy Lives project *	**Project webpage** https://www.hd4hl.org/prof-amos-laar-lauded-for-healthier-diets-for-healthy-lives-project/
11 Mar 2022	Ghana tackles non-communicable diseases through healthier diets	**Ghanaweb** https://www.ghanaweb.com/GhanaHomePage/NewsArchive/Ghana-tackles-non-communicable-diseases-through-healthier-diets-1488239
16 Mar 2022	Ministry of Health launches a 'double-duty policy bundle initiative' - Why Ghana embarks on such initiative	**Ghanaweb** https://www.ghanaweb.com/GhanaHomePage/NewsArchive/Ministry-of-Health -launches-a-double-duty-policy-bundle-initiative-Why-Ghana-embarks-on-such-initiative-1492034
12 Mar 2022	Kwaku Agyeman-Manu launches Healthier Diets for Healthy Lives Project to enhance health sector	**Ghanaweb** https://www.ghanaweb.com/GhanaHomePage/health/Kwaku-Agyeman-Manu-launches-Healthier-Diets-for-Healthy-Lives-Project-to-enhance-health-sector-1489115
11 Mar 2022	Suboptimal Diets contributes to various diseases-Hon. Agyeman Manu*	**Project webpage** https://www.hd4hl.org/suboptimal-diets-increases-the-risk-of-various-diseases-health-minister/
11 Mar 2022	Health Minister Launches HD4HL Project *	**Project webpage** https://www.hd4hl.org/health-minister-launches-hd4hl-project/
11 Mar 2022	Health Minister launches ‘Healthier Diets for Healthy Lives’ project*	**Project webpage** https://www.hd4hl.org/health-minister-launches-healthier-diets-for-healthy-lives-hd4hl-project-in-accra/
11 Mar 2022	Health Minister pledges gov’t’s commitment in improving Ghanaian food environment *	**Project webpage** https://www.hd4hl.org/health-minister-pledges-govts-commitment-in-improving-ghanaian-food-environment/
11 Mar 2022	Suboptimal Diets increases the risk of various diseases-Health Minister	**Project webpage** https://www.hd4hl.org/suboptimal-diets-increases-the-risk-of-various-diseases-health-minister/
13 Mar 2022	Health Minister Launches HD4HL Project | Health	**Ghana news updates** https://ghananewsupdates.com/health-minister-launches-hd4hl-project-health/
11 Mar 2022	Health Minister launches Healthier Diets for Healthy Lives Project in Accra	https://youtu.be/qiAX1LxVLqQ
11 Mar 2022	Launch of Healthier Diets for Healthy Lives (Hd4HL) Project	**Newswire GH** https://youtu.be/8ZhdYU4IjmY
11 Mar 2022	HD4HL project Launch in local language	**Peace FM** https://www.youtube.com/watch?v=ntOsKuHTJkQ
08 Feb 2023	A4H, HD4HL coalition applaud government’s fiscal policy approach to addressing unhealthy diets	**Metro TV** https://metrotvonline.com/a4hhd4hl-coalition-applaud-governments-fiscal-policy-approach-to-addressing-unhealthy-diets/
08 Feb 2023	A4H, HD4HL coalition applaud government’s fiscal policy approach to addressing unhealthy diets	**Original Tv** https://myoriginalonline.com/blog/a4hhd4hl-coalition-applaud-governments-fiscal-policy-approach-to-addressing-unhealthy-diets/
08 Feb 2023	A4H, HD4HL coalition applaud government’s fiscal policy approach to addressing unhealthy diets	**Ghanaweb** https://www.ghanaweb.com/vip/ernestsenanudovlo/A4H-HD4HL-coalition-applaud-government-s-fiscal-policy-approach-to-addressing-unhealthy-diets-51047
08 Feb 2023	A4H, HD4HL coalition applaud government’s fiscal policy approach to addressing unhealthy diets	**The Catholic Trend** https://catholic-trends.com/2023/02/08/a4hhd4hl-coalition-applaud-governments-fiscal-policy-approach-to-addressing-unhealthy-diets/
21 Feb 2023	HD4HL coalition welcomes government’s proposal to tax sugar-sweetened beverages	**Original TV** https://myoriginalonline.com/blog/hd4hl-coalition-welcomes-governments-proposal-to-tax-sugar-sweetened-beverages /
21 Feb 2023	HD4HL coalition welcomes government’s proposal to tax sugar-sweetened beverages	**Metro TV** https://metrotvonline.com/hd4hl-coalition-welcomes-governments-proposal-to-tax-sugar-sweetened-beverages /
21 February 2023	Advocating for Health Coalition lauds govt’s proposal to tax sugar-sweetened beverages	**Star FM** https://starrfm.com.gh/2023/02/advocating-for-health-coalition-lauds-govts-proposal-to-tax-sugar-sweetened-beverages /
21 February 2023	Health taxes a win-win strategy for Ghana—Coalition of researchers, CSOs	**Original TV** https://myoriginalonline.com/blog/health-taxes-a-win-win-strategy-for-ghana-coalition-of-researchers-csos/
21 February 2023	Health taxes a win-win strategy for Ghana—Coalition of researchers, CSOs	**Ghanaweb** https://www.ghanaweb.com/vip/ernestsenanudovlo/Health-taxes-a-win-win-strategy-for-Ghana-Coalition-of-researchers-CSOs-57461
21 February 2023	Health taxes a win-win strategy for Ghana—Coalition of researchers, CSOs	**Metro TV** https://metrotvonline.com/health-taxes-a-win-win-strategy-for-ghana-coalition-of-researchers-csos/

*The original link to these news items may no longer be active.

#### Position statements

The Coalition presented Position Statements to the Parliament of the Republic of Ghana, key Government MDAs, and to the President of Ghana. The Statements identified opportunities to strengthen the SSB tax proposal by Ghana. In table 2 below, we present page 1 of one the Postion Statements.

**Table 2:** Extracts from the Coalition’s Position Statement on SSB tax in Ghana (summary and opportunities to improve the proposed SSB tax Bill)

Joint Position Statement	
Implementation of taxes on sugar-sweetened beverages IN GHANA	
The Advocating for Health (A4H) and the Healthier Diets for Healthy Lives (HD4HL) Coalition.	
Accra, Ghana. 30 December 2022	
Summary	
A commendable move from the Government of Ghana	The Parliament of the Republic of Ghana recently debated, voted, and approved a Bill to tax sugar sweetened beverages (SSBs) and other commodities —as per the.Ghana Excise Duty Amendment Bill, 2022 We commend the Government of Ghana for proposing the policy, as health costs and deaths linked to diet-related non-communicable diseases (NCDs) mount.
Diet-related NCDs are serious health issues in Ghana	Several local studies report a high prevalence of overweight/obesity among Ghanaian children and adults. Ranging from 16% to 46% (for children aged 6–15 years) and 25% to 47% (for adults aged 15 years or older), the prevalence is significantly higher in women vs men, and in urban vs rural Ghana. One study found close to 50% of adults with diabetes to be overweight or obese. People (particularly children) who suffer from overweight or obesity have an elevated probability of developing other NCDs such as type 2 diabetes, hypertension and stroke in later life.
SSBs are significant contributors	The determinants of obesity are many. However, dietary factors such as excessive consumption of calorie-dense, nutrient-poor foods such as SSBs are the most important. SSB consumption is also implicated in other diet-related NCDs such as dental caries. Data from jurisdictions across the world that have enacted SSB taxes correlate the implementation of such taxes with decreased consumption of SSBs.
Ghana ExciseDuty Amendment Bill, 2022 could save lives	A successful implementation of the Excise Duty Amendment Bill, 2022—proposed by the Government of Ghana to, among other items, impose taxes on SSBs, could have significant positive health impacts for Ghanaians.
But there are gaps to be addressed	While commending the Government of Ghana for proposing the policy, the Advocating for Health (A4H) and Healthier Diets for Healthy Lives (HD4HL) Coalition reaffirm their concerns about the harms caused by SSB consumption, they outline gaps in the proposed policy, and offer recommendations for the Government of Ghana to strengthen the policy.
What we recommend	We recommend the adoption of a sugar content-based specific excise tax and earmarking of accrued revenue to health promotion interventions including health research, or supporting social protection programmes such as the National Health Insurance Scheme (NHIS), the Ghana School Feeding Programme (SFP) or the Livelihood Empowerment Against Poverty (LEAP) Programme. Such arrangements promote tax equity and health equity. 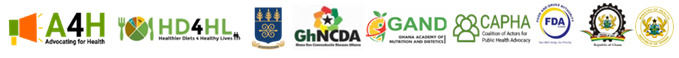

Source: The A4H Coalition.

NCD, non-communicable disease.

#### Press statements

Whenever the Coalition deemed it necessary, press statements were issued.

#### Press conferences

A Press Conference on Health Taxes was organised to among others, drum home the message that ‘Taxation of SSBs is a Win-Win-Win Strategy for Public Health, for Government Revenue, and for Health Equity’

#### Stakeholder engagements and dissemination activities

We actively participated in and contributed to relevant local, regional and international events (including international conferences and seminars) where the Coalition’s work and experiences were disseminated. We produced policy briefs, information briefs and advocacy materials, and used them to engage various stakeholders.

#### Blogs, op-eds and social media

Coalition members wrote blogs (eg, https://www.alaar.org/my-take-on-matters-that-matter-articles/), op-eds (see table 1) and kept active online presence (for example, on Project website—https://www.advocating4health.org/, and on social media).

## What we have achieved thus far

Owing in part to the Coalition’s food advocacy and activism, there is currently a national awareness that development and implementation of healthy food environment policies will make unhealthy foods unaffordable, unavailable and unattractive. This food activism work has valorised and increased demand for such policies in Ghana. Currently, the Ghanaian government is working with other actors to deliver a fit-for-local purpose nutrient profiling system as well as four double-duty food-based policies. The policies in the bundle include front-of-pack nutrition labelling policy, policy restricting child-directed marketing of unhealthy foods, food-related fiscal policy and public food procurement and service policy.

Taken together, our food activism work has contributed to creating a favourable environment for food-related health taxes in Ghana. At the time of writing of this paper, the government of Ghana had demonstrated resolve to roll out some of the policies. The Parliament of the Republic of Ghana in December, 2022, and in March 2023, voted to amend the Ghana Excise Duty Act, 2014 (ACT 878). The amendments include imposition of excise duty (20 per centum of the ex-factory price) on sweetened beverages including fruit juices (eg, grape and vegetable juices unfermented and containing spirits whether or not containing added sugar of other sweetening matter). Other products covered by the Act include cigarette and tobacco products, wines, malt drinks and spirits, plastics and plastic products. On 3rd April 2023, the President of the Republic of Ghana signed the approved Excise Duty Amendment Bill of 2022 into law (Act 1093).^37^

The Coalition will continue to generate the needed evidence, will curate and avail the evidence for advocacy, and scholar activism toward convincing the government of Ghana (and others in sub-Saharan Africa) to implement comprehensive policy measures that make unhealthy foods unattractive and unavailable. To advocate to any government for healthy food policy is to recognise and respect the mandate of the government. The role of governments in protecting, promoting and assuring the health of their citizens is primarily grounded in national legislations, policies and international conventions. One vital step in making this right of citizens realised is to make our food environments more equitable, more ethical and more democratic.

## Conclusions

We aimed to present the A4H Coalition as a best practice example of how coalition building, evidence-informed advocacy and scholar activism can valorise and increase demand for healthy food policies. While this is not a magic bullet, we believe that well thought-out coalitions that are armed with context-relevant data and capacitated to advocate for the health of their countries may make food-related health taxes palatable in their jurisdictions.

**Figure 3:**
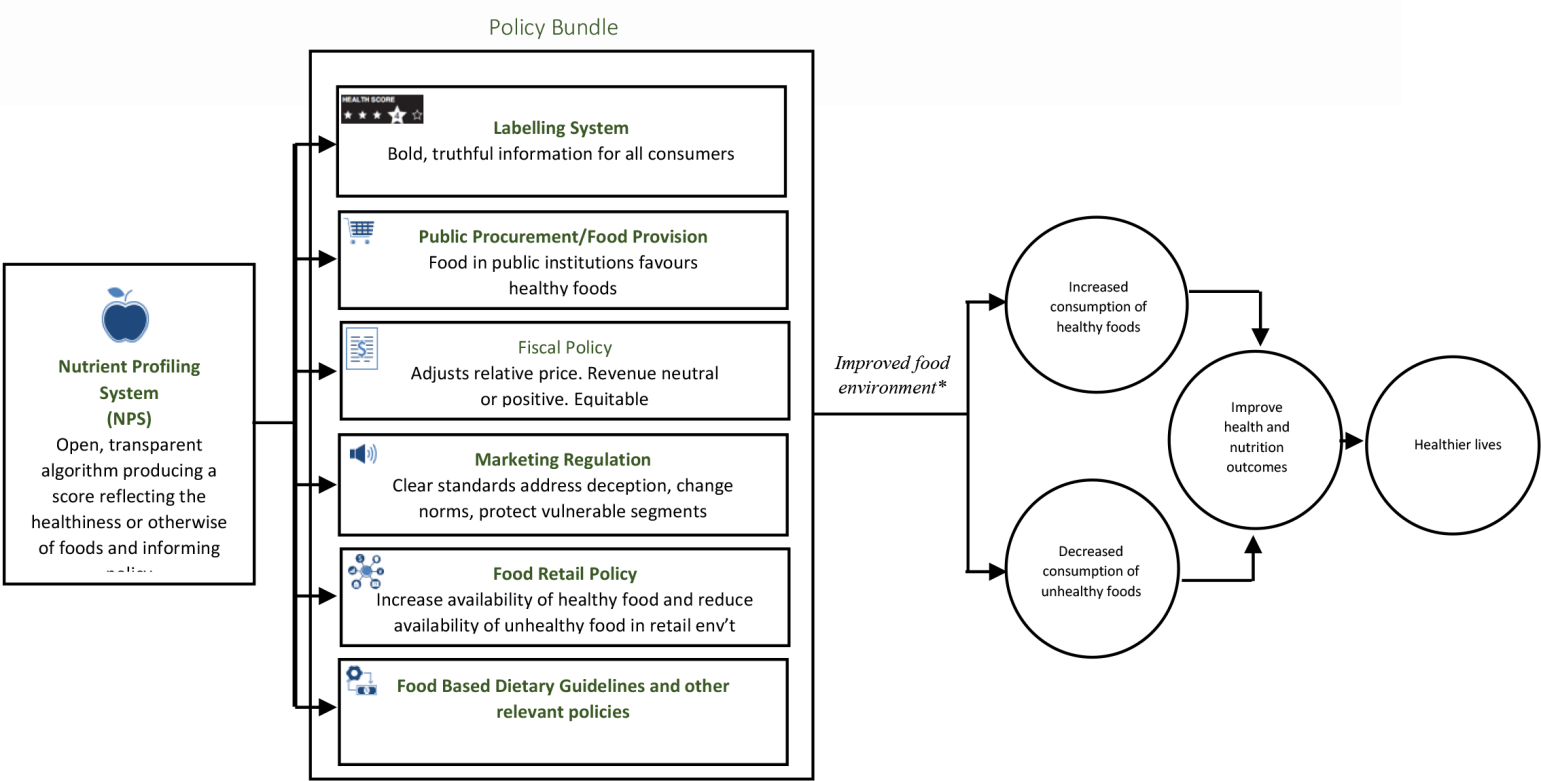
Double-duty food-based policy bundle to assure healthier diets in Ghana. Source: The HD4HL Project.

It is worthy of note that this is particularly challenging in the African context. As noted earlier, power asymmetries between ‘public’ and ‘private’ interests confound promulgation and implementation of these policies.^38 39^ Public health advocates have to be willing and ready to counter and debunk oppositional arguments from actors with vested interests. Advocates need to appreciate the peculiar heterogeneity of their food environments and its variegated political economies. Facing a syndemic of undernutrition, overweight/obesity and other diet-related NCDs, enforcing policies that dis-incentivise consumption of unhealthy diets is not enough. There must be complementary policies that avail healthy/nutrient-rich foods. Thus, a policy bundle comprising low agency and high agency policy interventions^40^ that inform and empower; guide and influence; those that dis-incentivise consumption of unhealthy diets as well as those that avail and incentivise consumption of healthier diets are needed in the African setting. Thus, such policies/guidelines as the food-based dietary guidelines which centres and celebrates whole and minimally processed foods, the four low and high agency policy interventions outlined in figure 3 and others needed to be complementary and not antagonistic. Finally, appropriate evaluation of the policy implementation is needed. Limited data, and lack of monitoring and enforcement capacity may provide opportunities for industry to selfishly game the system. The policies need to be enforced, monitored and evaluated. Should such policy evaluations recommend amendment of the policy, governments should not hesitate to explore the ample space available to adjust the tax design to further improve their impact on health.

## Data Availability

Data available upon reasonable request.
